# The Role of Accreditation in Supporting Educational Quality During Difficult Circumstances

**DOI:** 10.15694/mep.2017.000052

**Published:** 2017-03-16

**Authors:** Barbara Barzansky, Robert Hash

**Affiliations:** 1 LCME

**Keywords:** Accreditation, Quality Assurance

## Abstract

This article was migrated. The article was marked as recommended.

Accreditation aims to ensure that generally-accepted standards of educational program quality are being met. The Liaison Committee on Medical Education (LCME), the accrediting body for medical education programs leading to the MD degree, has a process to address circumstances at medical schools arising from acute emergency situations. This involves consultation with faculty and administration at the affected school and collection of data related to compliance with accreditation standards through written reports and on-site visits. The actions of both the medical schools in New Orleans and the LCME following Hurricane Katrina in 2005 illustrate this process. While the need to respond to requests for information from an accrediting body places additional burdens on a medical school’s faculty and administration during an emergency, such oversight also ensures that the medical education program continues to meet standards. This provides assurance of educational quality to school personnel and students, as well as to the school’s publics. Accreditation agencies should consider how their standards and processes support a timely and flexible response to disaster situations that occur at accredited schools.

## Introduction

An early definition of accreditation [ Blauch, 1959] includes the elements that remain relevant today: “Accreditation.. is the recognition accorded to an institution that meets the standards or criteria established by a competent agency or association. Its general purpose is to promote and insure high quality in educational programs (page 3).”

The key concepts included explicitly or implicitly in the definition are that accreditation is standards-based and that it aims to ensure educational program quality. Both these concepts are centrally important when medical schools find themselves in difficult circumstances. In such situations, accrediting bodies must consider what it means to be in compliance with standards in the context of medical school adaptations to their changed circumstances.

In this commentary, we use the Liaison Committee on Medical Education (LCME) as an example of an accrediting body that quickly responds to information that a medical school is currently in difficult circumstances. The LCME has policy that requires it to do so and procedures to follow to evaluate the impact of the circumstance on the compliance of the medical education program with accreditation standards [ LCME, 2016].

The LCME currently accredits the medical education programs leading to the MD degree in the United States and, in conjunction with the Committee on Accreditation of Canadian Medical Schools, the 17 medical schools in Canada [ LCME, 2017]. Medical education programs are reviewed by the LCME on an eight-year cycle. The review is based on accreditation standards that apply to the year in which the review takes place. For the 2016-2017 academic year [ LCME, 2015], there are 12 standards with 93 associated elements. Accreditation standards and elements address categories such as medical school leadership and administration, educational program objectives, curricular content, management of the medical educational program, student services, educational and clinical facilities, finances, and faculty numbers and availability to teach.

In addition to regular reviews, the LCME requires medical schools to promptly submit a notification of circumstances that might affect the ability to carry out the medical education program. This may involve a need for additional resources, such as an imminent class size increase, or the loss of important resources, such as clinical teaching sites [ LCME, 2016]. Notification allows the LCME to review in real time that there is an appropriate balance between student enrollment and the constellation of resources to support the cohort of enrolled students. If the LCME learns of changed circumstances either directly from the school or through information in the press, it may request a written report from the school, schedule a focused survey visit to review the specific circumstances, and/or direct the LCME Secretariat to conduct a consultation visit or Secretariat fact-finding visit (a form of focused visit). The four professional members of the LCME Secretariat who carry out consultations and make fact-finding visits are experienced medical educators who regularly participate in full accreditation reviews.

## Categories of “Difficult Circumstances”

There are two general categories of difficult circumstances:

### An Unanticipated Emergency

An acute emergency can affect the ability of one or more medical schools in a region to function. Examples of acute emergencies include a natural disaster (e.g., hurricane, earthquake, flooding) or an outbreak of war or civil unrest. In this category, a medical school must identify the strategies and resources to rapidly adjust to the new circumstances. After the emergency, the medical school returns to “normal” or to a new steady-state after the disruptions caused by the emergency have been resolved.

### Ongoing Difficult Circumstances

In this category, there are circumstances, such as financial or other resource limitations, that affect the ability of a medical school to meet the requirements of the accreditation standards on an ongoing basis. For example, limited medical school funding could make it difficult to recruit and retain faculty or renovate/construct education facilities, which, in turn, affects the ability to deliver the medical education program. Unless the causative circumstances improve, the situation will not be resolved.

These categories may overlap, in that an acute emergency may develop into a long-term situation if the needed resources and strategies to address the emergency are not available or the emergency itself is prolonged.

This commentary focuses on how the LCME, as an accreditor, responded to an acute and unanticipated emergency. Ongoing difficult circumstances tend to be addressed through regular accreditation reviews or focused reviews resulting from events reported by the program or contained in the press or in other public sources of information.

## The LCME Response to an Acute Emergency

On August 29, 2005, Hurricane Katrina passed east of New Orleans, Louisiana, USA and resulted in widespread flooding when the levees protecting the city failed [ Taylor, 2007]. The functioning of medical schools within the city were disrupted, due to damage to their educational and research facilities and their hospitals; loss of transportation and civil services; loss of utilities; lack or food and clean water; and general displacement of the population, including faculty and students. The responses of the medical schools and of the LCME to the aftermath of Hurricane Katrina is an illustration of how the accreditation system intervened during an acute emergency affecting the short-term functioning of these medical schools.

### What the Medical Schools Did

There were two medical schools and their associated teaching hospitals that were affected by Hurricane Katrina. The specific steps taken by each medical school were based on the individual school characteristics, but the strategies were similar and resulted in quick responses and timely resolution.

The Louisiana State University School of Medicine - New Orleans (LSU-NO) evacuated students, administrators, and faculty to Baton Rouge, an existing organizational component of the medical school and an existing educational site for medical students that is located outside the region of storm damage. Education for students in the pre-clerkship (basic science) phase of the curriculum began by the end of September 2005. A temporary faculty research infrastructure was created through shared resources with LSU faculty at the Baton Rouge site and faculty at other institutions. New housing for faculty and students was organized. Renovations at the New Orleans campus were sufficiently complete to allow the basic science portions of the curriculum to resume within about nine months. [Hollier, 2006; Wikipedia, 2017]

Tulane University School of Medicine relocated its pre-clerkship students and teaching staff to the Baylor College of Medicine in Houston, Texas. Students in the clinical years did their clerkships at teaching hospitals associated with several Texas medical schools. Students and faculty returned to New Orleans during 2006 [ Wikipedia 2016].

In summary, both medical schools re-located administrators and faculty to temporary facilities outside New Orleans so that they could continue to provide the medical education program. Those facilities were either within the medical school system (LSU-NO) or external to it (Tulane). Much of the medical education program of each school returned to New Orleans within the year.

### What the LCME Did

LCME records describe that information was collected from both New Orleans medical schools during the year following Hurricane Katrina. The requested information focused on the availability of resources to implement the medical education program during the emergency, including such things as access to educational and clinical facilities, availability of medical school faculty to teach the program’s students, and access of students to a wide range of student services. The need to have these resources available to all students is codified in accreditation standards.

The LCME follow-up included both a request for written information and on-site review. A written report on the move to Baton Rouge was requested from LSU-NO by the end of November 2005. This was followed by an additional written report in May 2006 and an on-site visit to Baton Rouge in September 2006. Similarly, the Tulane School of Medicine clinical teaching sites in Texas were visited in November 2005, a written report from the school was due in May 2006, and an on-site visit to the New Orleans campus was conducted in August 2006. Each of the written and survey visit reports were reviewed by the LCME to ensure that compliance with relevant accreditation standards was being maintained.

### A Medical School’s Perspective on the LCME’s Actions

To obtain a school’s perspective on the role accreditors can play under difficult circumstances, the authors interviewed Dr. N. Kevin Krane, who served as vice dean for academic affairs at Tulane University School of Medicine at the time Hurricane Katrina struck, and was a central figure in the coordination of the school’s response to the emergency. Dr. Krane stated: “The LCME was very helpful in making sure that in the process of responding we protected the integrity of the curriculum for the benefit of the students. They gave us the parameters by which to operate under the special circumstances. It was important to know that our actions would be supported. The LCME’s flexibility gave us the authority to move forward. Without the flexibility, we would have had to shut down the program.”

Dr. Krane noted that early and frequent communication between the program and the LCME was essential. His recommendation for accreditors with programs in the midst of an emergency circumstance is to for the accreditor to reach out early, asking it can support the needs of the program. “Communication is essential, so that everyone is on the same page, and the accreditor isn’t seen by the program as an inflexible barrier, and the program isn’t viewed by the accreditor as taking unreasonable actions.”

## Accrediting Body Oversight

Unanticipated emergency situations place a significant burden on medical school faculty and administrators, since they must react swiftly to develop and implement plans to adapt to the changed circumstances. As in the example described above, displacement of the medical education program and disruption of basic services required that new educational and clinical facilities be identified, infrastructure such as information technology be developed and deployed in new locations, and student and faculty support mechanisms be identified or created. This must happen quickly in the context of unfamiliar surroundings. Students may be particularly vulnerable in emergency situations. In addition to the disruption to their educational program, students may face isolation and separation from their support systems, including faculty mentors and access to health and psychological services; harbor anxiety regarding the potential effects of the disruption on their standardized test scores and letters of recommendation; and have worries about the financial implications of their uncertain circumstances.

In this context, it is important to consider that there are burdens and benefits associated with accreditation oversight in difficult emergency situations. An additional effort is required of tired and overworked faculty and administrators, and sometimes of students, in responding to requests for information and meeting with accreditation reviewers during visits. This additional effort may make members of the medical education community feel overburdened and may take time away from activities that would aid their adjustment to the difficult situation. Also, accreditation standards may force, or be perceived as forcing, medical schools to follow a path that is not optimal in the context imposed by the emergency situation.

However, close oversight by an accrediting body can provide some benefits. Consultations by accrediting agency staff may help keep a disbursed faculty and student body connected to each other and to the national medical education community. Having to consider and report on how the school is remaining in compliance with standards could ensure that important areas, such as student services, are not forgotten in planning for the new situation. Also, a positive report from an accrediting body reassures faculty, students, and the public that the medical school still is delivering a quality medical education program, even if there are variations from how the program was conducted prior to the emergency.

## Take Home Messages for Accrediting Bodies

Schools facing unanticipated emergencies must be flexible yet rapidly responsive to facilitate continuation of the educational program and ensure that students are appropriately supported. Accrediting bodies should behave similarly. Accreditation remains responsible for judging program quality, so that desired student outcomes are achieved and, in the case of medical education, the public is protected. The interests of institutions and learners may be best served through adaptation and flexibility in application of the accreditation process and its standards. There should be particular attention to whether and how schools are continuing to address the support needs of students - educational, physical, and emotional.

Certain actions by accrediting bodies may help prepare them to respond when a medical school faces an unanticipated emergency. The following suggestions, based on the typical LCME process, may be of use.

### Adopting Relevant Accreditation Standards

Accrediting bodies should have a requirement that medical schools have a disaster plan. While a plan may not address all contingencies, developing and updating a plan keeps the possibility of an unanticipated emergency in the minds of medical school planners and others who may be required to take action should an emergency occur.

### Providing Consultation and Support

In emergency situations, accrediting body staff members need to be available for consultation to schools to assist them in interpreting how to apply accreditation standards to their specific circumstances and to the plans that they are developing and implementing to respond to the emergency.

### Interpreting the Intent of Accreditation Standards

Accrediting agencies need to exhibit short-term flexibility in permitting temporary solutions that address the intent of the accreditation standards and also should be receptive to creative solutions to demonstrate compliance. Implementation of this suggestion depends particularly on the structure of standards. Quantitative standards can be prescriptive and not allow deviation from the specified benchmark, while more qualitative standards can allow a more flexible judgment of compliance. In either case, accrediting agencies need to consider the amount of deviation that will be acceptable.

### Setting Benchmarks

Determining the amount of acceptable deviation could be facilitated by having quantitative or qualitative benchmarks for compliance with standards. Examples of quantitative-type benchmarks include the percent of students passing a national examination or the percent of students being observed performing a physical examination during clerkships. Such quantitative benchmarks typically represent the minimal acceptable level. Qualitative benchmarks would be represented by the presence of expected elements in a process. For example, in a standard related to academic counseling, the minimal acceptable process could include having a system to identify students in academic difficulty, personnel to provide academic support and tutoring, and mechanisms to determine if a learning disability exists. For qualitative benchmarks, there may be multiple ways to meet the expectations of the standard.

In summary, accrediting bodies should explicitly plan for how they might address an emergency situation at an accredited program. This requires developing standards, policy, and procedures.

## Notes On Contributors

Barbara Barzansky, PhD, MHPE serves as the Co-Secretary of the Liaison Committee on Medical Education, based at the American Medical Association

Robert Hash, MD, MBA serves as the Assistant Secretary of the Liaison Committee on Medical Education, based at the American Medical Association.

## Appendices

None included.

The opinions expressed in this article are those of the authors and do not constitute the policy of the LCME.
