# Supplementary material for: The Role of Accreditation in Supporting Educational Quality During Difficult Circumstances
Source: MedEdPublish (2016). 2017 Mar 16;6:52. [Version 1] doi: 10.15694/mep.2017.000052 (PMC10885244; doi:10.15694/mep.2017.000052)
Supplement: Peer review history

# Reviewer report: Reviewer response for version 1

**DOI:** 10.21956/mep.19465.r28699

**Published:** 2017-03-20

**Authors:** Peter Dieter

**Affiliations:** 1 AMSE

This review has been migrated. The reviewer awarded 5 stars out of 5 An excellent and well written paper giving examples how the LCME responses to difficult circumstances (unantipicitaded or ongoing). The paper shows that the medical school has its own responsibility to react to those circumstances and the accreditation agency has to be flexible in order to support the school, teachers and students and guarantee an ongoing good education for medical students. Other accreditation agencies should have a similar policy.

Reviewer Expertise:

NA

No decision status is available

# Reviewer report: Reviewer response for version 1

**DOI:** 10.21956/mep.19465.r28696

**Published:** 2017-03-19

**Authors:** Michelle McLean

**Affiliations:** 1 Bond University

This review has been migrated. The reviewer awarded 5 stars out of 5 Thanks for this well-written article on the role of the LCME as an accrediting body for medical schools in crisis (unanticipated and ongoing) to not only ensure the programme continues but also to take care of students and staff well-being. The take-home messages are important, particularly messages 1 and 2. One relates to each medical school having contingency plans. This will become increasingly important in terms of increasingly unpredictable weather events (an effect of climate change?) and when students are on international electives. The second take-home point that is important is the need for early notification. This article provides guidance for fledging accreditation bodies and should also alert medical school leaders to the need for a range of contingency plans.... What if...? scenarios.

Reviewer Expertise:

NA

No decision status is available

# Reviewer report: Reviewer response for version 1

**DOI:** 10.21956/mep.19465.r28697

**Published:** 2017-03-16

**Authors:** Dujeepa D. Samarasekera

**Affiliations:** 1 National University of Singapore

This review has been migrated. The reviewer awarded 4 stars out of 5 Good insights into accreditation processes. Expert commentry. Useful for medical school faculty and administrators involved in maintaining QA processes.

Reviewer Expertise:

NA

No decision status is available

# Reviewer report: Reviewer response for version 1

**DOI:** 10.21956/mep.19465.r28698

**Published:** 2017-03-16

**Authors:** Trevor Gibbs

**Affiliations:** 1 AMEE

This review has been migrated. The reviewer awarded 4 stars out of 5 An interesting and well written paper that describes how an accreditation procedure can be modified during a difficult circumstance.I feel that the paper provided some answers and opportunities to both institution and accrediting body, many of which I feel are useful to those who are involved in accrediting processes- rather than placing a halt on the accreditation process those involved found a satisfactory and effective way around the problem, whilst maintaining credibility and safety. It would be interesting to compare how other accrediting bodies respond to such difficult circumstances

Reviewer Expertise:

NA

No decision status is available
